# Effect of thermal and mechanical cycles on shear bond strength of zirconia core to porcelain veneer under different surface treatments

**DOI:** 10.15171/joddd.2019.035

**Published:** 2019-10-07

**Authors:** Tahereh Ghaffari, Elnaz Moslehifard, Mehrnaz Motiei

**Affiliations:** ^1^Department of Prosthodontics, Faculty of Dentistry, Tabriz University of Medical Sciences, Tabriz, Iran

**Keywords:** Air abrasion, mechanical cycle, porcelain veneer, thermal cycle, zirconia

## Abstract

***Background.*** Due to the fragile nature of all-ceramic restorations, it is necessary to provide an appropriate (core) infrastructure to support the veneering porcelain. The veneer detachment and chipping are disadvantages of these restorations. Several techniques have been proposed to minimize these problems. This study evaluated the effect of thermal and mechanical cycles on the shear bond strength of zirconia core to porcelain veneer under different surface treatments.

***Methods.*** Sixty disk-like zirconium samples were randomly divided into three groups. The first group was polished and veneered with porcelain, without additional surface treatments. The two other groups were subjected to different surface treatments (modified aluminum oxide by silica and activator‒aluminum oxide and primer) and veneering with porcelain. Half of the samples in each group were subjected to 6000 thermal cycles and 20,000 masticatory cycles of 50 N to imitate the intraoral conditions; the other half were placed in distilled water at 37°C until the shear strength test. Each sample was then buried using PMMA in a mounting jig so that the gap between the core and the veneer could be placed upward. Then, they were exposed to shear stress using a universal testing machine at a rate of 1 mm/min until fracture. The maximum force leading to the fracture was recorded.

***Results.*** Comparison of the groups showed that the highest shear bond strength was related to the samples treated with aluminum oxide and primer, without applying thermal and masticatory cycles, which indicated no significant difference from the group treated with aluminum oxide and primer, with thermal and masticatory cycles. The lowest shear bond strengths were related to the polished samples without surface treatment by applying thermal and masticatory cycles (P=0.001), which indicated no significant difference from the untreated group without thermal and masticatory cycles.

***Conclusion.*** Based on the results, treatment with aluminum oxide and primer increased the shear bond strength of zirconia core to porcelain veneer. Thermocycling and masticatory cycles failed to reduce the shear bond strength in all the three groups significantly

## Introduction

With the advances in science and technology, all-ceramic restorations have become very popular in contemporary dentistry.^1^ Due to the fragile nature of all-ceramic restorations, it was necessary to use an appropriate (core) infrastructure for supporting the veneering porcelain until zirconia was introduced as a suitable core for all-ceramic restorations in the 1990s.^2^ The pure zirconia material is unstable in the tetragonal form at room temperature. Therefore, it is used to stabilize yttrium oxide and magnesium oxide. Zirconium, which is used today, contains yttrium oxide with a molar percentage of 3%.^3^ The advantages of zirconia as a core material lie in its mechanical properties. Among all the dental ceramics, zirconia has the highest flexural strength and the highest fracture resistance. Its resistance to pressure is 2,000 MPa.^4^ Another feature of zirconia is that it can change to monolithic phase under the stress of the tetragonal phase. Following this deformation, a volume increases of 4% will occur, causing shrinkage stress in zirconia, which prevents the crack from spreading, referred to as transformation toughening.^5^

One of the disadvantages of zirconia-based restorations is related to its opaque nature. Although the zirconia frameworks are more beautiful than the metal ones, they are very white and opaque. In all-ceramic zirconium systems, this core is constructed by a special CAM process, and then the resultant core is veneered by typical porcelains, using the layering and/or pressing techniques.^6^ The zirconia core provides excellent support for the veneering porcelain.^7^ Another problem is detachment and chipping of the porcelain veneer.^6^

However, factors such as the veneering porcelain thickness, limitations of the veneering porcelain bond with zirconia core, the weak nature of the bond, the low support of the covering ceramic, false framework design, direction, intensity and the number of occlusal forces, imperfections in the ceramics and the remaining stresses due to the thermal expansion coefficient differences and poor wettability of the core by the porcelain might cause porcelain delamination and zirconium core exposure or porcelain veneer chipping, disrupting the zirconium fixed prostheses treatment.^8^ Since the zirconia core is commonly shaped in the form of a uniform core layer in the available all-ceramic systems, veneered porcelain has different thicknesses in different areas. As a result, it will chip faster and eventually fracture under bending forces.^9,10^

Devigus et al^11^ indicated that the fracture in the veneered zirconia crowns mostly occurs as veneer detachment from a sound core, while fracture happens both in the core and veneer in the veneered lithium disilicate crowns. Some reported a fracture of 3% to 8%.^12^ In addition, some researchers reported the prevalence of porcelain chipping as follows: 15% after 24 months; 25% after 31 months; 8% after 32 months; and 13% after 388 months.^13^ The prevalence of this type of fracture is considerably higher than that of metal‒ceramic prostheses (0.4% for single crowns and 2‒44% for fixed prostheses in 5 years).^3^ Ozkurt et al^14^ investigated the effect of ceramic coating on the shear bond strength of 4 business types of zirconia and showed that zirconia type has a significant effect on the bond strength, and the core-veneer bond depends on the type of materials.

Another study showed that the bond between zirconia and ceramic veneer is chemical, with 19.3 MPa for zirconia and 8.22 MPa for ceramic,^15^ while another report indicated that this bond is mechanical.^2^ Melo et al^16^ measured the amount of ceramic endurance which was 518 N for aluminum restorations, 282 N for lithium disilicate, and 755 N for zirconia. Sailer et al,^17^ in their laboratory study, showed that the zirconia-based fixed prostheses had a fracture resistance of more than 20,000 N.

Rocatec and air abrasion + liner were proposed as the best methods for improving the shear bond strength of porcelain veneer in studies by Minori and Musharraf.^18^ Therefore, in similar studies, there are deficiencies such as lack of examination of the shear bond strength, a small number of samples, lack of a simultaneous study on both different treatment methods, including Rocatec and air abrasion + liner, or lack of examination of the shear bond strength under both the mechanical and thermal cycles simultaneously.^19^

Thus, mechanical and thermal tests were evaluated in the same oral conditions in order to validate a clinical approach before it was proposed. In the present study, we compared the best methods of shear bond strength. Since porcelain bonding to the framework is the key factor in the successful performance of two-layer veneer-framework restorations, the shear bond strength was considered in the present study. The present study was conducted based on the hypothesis that "the shear bond strength is not different in the three methods of veneering zirconia base with and without mechanical and thermal cycles."

## Methods

**Figure 1:**
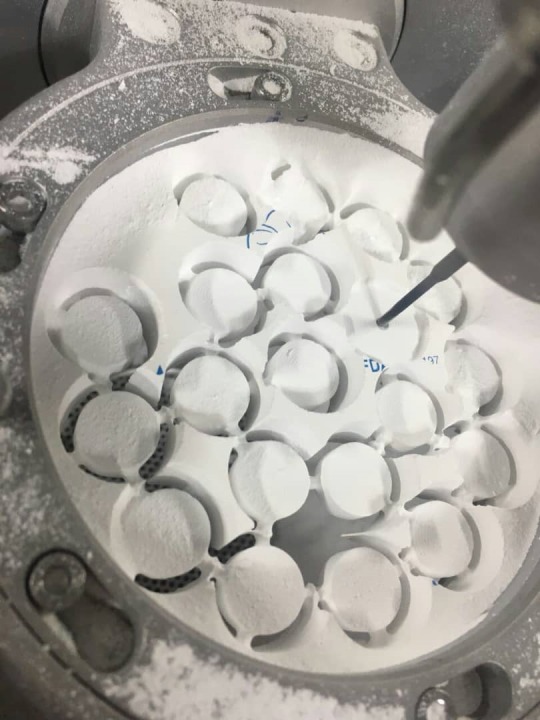
Making zirconia using the CAD-CAM system.

**Figure 2:**
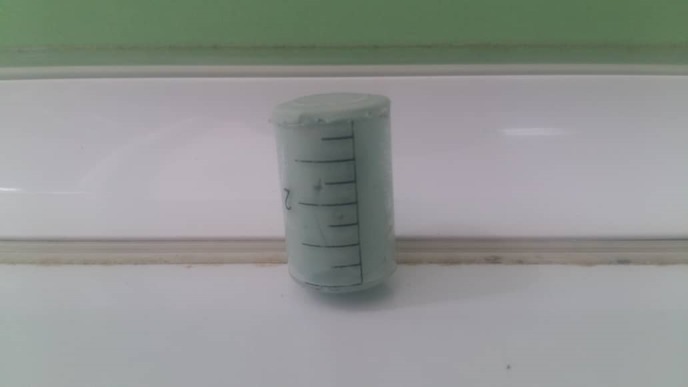
Zirconia core inside the putty mold.

In the present in vitro study, 60 disk-like polished zirconia samples (Dentium, The Rainbow™ Shine-T Zirconia Block, Korea, Seoul), 10 mm in diameter and 4 mm in thickness, measured by a digital micrometer (Absolute 500, Kitutoyo Co, Aura, IL, USA, Washington, DC), were prepared by the CAD-CAM device (Figure 1). ISO specifications for preparing the samples for ceramic were from Kaplan et al^20^ (2015) study. All the disks were sintered in the furnace (Cercon Heat furnace, Degu Dent Co, Germany, Hanau) for 10 hours at 1550°C to reach a full density. The surface of the samples was polished in one direction using #600 silicon carbide paper (Carbide Silicon P, MATADOR Co., Germany, Berlin), and cleaned using an ultrasonic device (Biosonic, Waledent Co., Berlin, Germany) containing acetone (Acetone, Aylar Co., Iran, Tehran) and distilled water (Deionizer Device, 200, Pars Kimiya Mavad Co., Tehran, Iran) for 15 minutes. The samples were then buried using a cylindrical mold (disposable syringes) (Helal Medical Equipment Company, Tehran, Iran) in a putty condensation silicon (Putty, Coltene Co., Berne, Switzerland) (Figure 2).

For surface treatment, the samples were randomly divided into three groups:

**Group 1:** Polished samples as the control group (NMNT and NMT)

**Group 2:** Application of tribochemical air abrasion using the Rocatec system (3M ESPE, Washington DC, USA). The air abrasion procedure was conducted using a sandblasting machine, using silica powder with aluminum oxide for 13 seconds at an air pressure of 2.8 bar at a distance of 10 mm from the zirconia surface, immediately followed by the application of the activator (Clearfil porcelain bond activator, Yahishama Co., Tokyo, Japan) on the samples (SMT and SMNT).^21^

**Group 3:** The samples were mounted in a special holder at a distance of 15 mm from the surface and the tip of the air abrasion machine (Kavo EWL, Type 5423, Biberach Co., Berlin, Germany); the aluminum oxide particles, measuring 110 µm, were applied with a pressure of 3.5 bar for 5 seconds.^22^ Then, the primer (GC Corporation, Yahishama Co., Tokyo, Japan) was uniformly applied to the samples (PMNT and PMT). After surface treatments and separation from the mold, the porcelain was baked on the samples by a plastic ring measuring 1 mm in diameter and 2 mm in height. The conditions for baking the veneers on the base of zirconia were as follows: pre-heat: 55ºC; holding time: 5 minutes; final temperature: 99ºC; heating rate: 55ºC; drying time: 90 seconds.

Half of the samples in each group were subjected to thermal and mechanical cycles to imitate the intraoral conditions. First, the samples were placed under 6000 thermal cycles at 5‒55°C in deionized water with a 15-second dwell time and a transfer time of 5 seconds. The ISO value for thermal cycles was from a study by Morresi et al.^23^ Then, they were fixed with self-curing acrylic resin. Thereafter, they were veneered by 20,000 cycles of 50-N force perpendicular to the surface of porcelain (Cyclic Loading SD, Mechatronik Chewing Simulator CS4, Minerline Co., Berlin, Germany), and applied at a frequency of 1 cycle/s.^24^

The remaining samples were immersed in distilled water at 37°C until the shear bond strength test. Then, each sample was exposed to shear stress from the porcelain and zirconia substrate region, using a universal testing machine (TLCLO, Dartec Ltd., Stourbridge Co., London, England) at a rate of 1 mm/min until fracture. The maximum force leading to the fracture was recorded.

Three types of adhesive, cohesive and mixed fractures were visible based on the fracture site. The results were reported using descriptive statistical methods (means, standard deviations, and percentage frequencies). Independent t-test was used to compare shear bond strengths with and without thermal and mechanical cycles. One-way ANOVA was used to compare the shear bond strength between two types of surface treatment and one group without surface treatment. Two-way ANOVA was applied to compare the shear bond strength concerning the presence or absence of surface treatment and mechanical and thermal cycles. The significance level of P<0.05 was considered, and statistical analyses were performed using SPSS 17.

## Results

In the present study, 60 samples of zirconia were evaluated in six groups. The mean ± standard deviation of shear bond strength in the NMT group was 15.96±14.04 MPa, with 16.15±11.42 MPa in the NMNT group. Comparison of the shear bond strength of the polished samples with and without thermocycling and masticatory cycles indicated no significant differences (P=0.974) (Table 1).

**Table 1:** Comparison of shear bond strength of the polished samples with and without thermocycling and masticatory cycle

**Group**	**Number**	**Mean ± SD (MPa)**	**Sig.**
NMT	10	15.96 ± 14.04	0.974
NMNT	10	16.15 ± 11.42	

The mean ± standard deviation of the shear bond strength in the SMT group was 16.65±9.03 MPa, with 17.88±13.08 MPa in the SMNT group. Comparing of the shear bond strength of the samples treated with the modified aluminum oxide by silica and activator, with and without thermocycling and masticatory cycles, showed no statistically significant difference (P=0.712) (Table 2).

**Table 2:** Comparison of shear bond strength of the samples treated with the modified aluminum oxide by silica and activator with and without thermocycling and masticatory cycle

**Group**	**Number**	**Mean ± SD (MPa)**	**Sig.**
SMT	10	16.65 ± 9.03	0.810
SMNT	10	17.88 ± 13.08	

The mean ± standard deviation of shear bond strength in the PMT group was 19.38±12.99 MPa, with 20.58±11.68 MPa in the PMNT group. However, no significant difference was observed in the shear bond strength between the two groups treated with aluminum oxide and primer, with and without thermocycling and masticatory cycles (P=0.830) (Table 3).

**Table 3:** Comparison of shear bond strength of the samples treated with aluminum oxide and primer with and without thermocycling and masticatory cycles

**Group**	**Number**	**Mean ± SD (Mpa)**	**Sig.**
PMT	10	19.38 ± 12.99	0.830
PMNT	10	20.58 ± 11.68	

**Figure 3:**
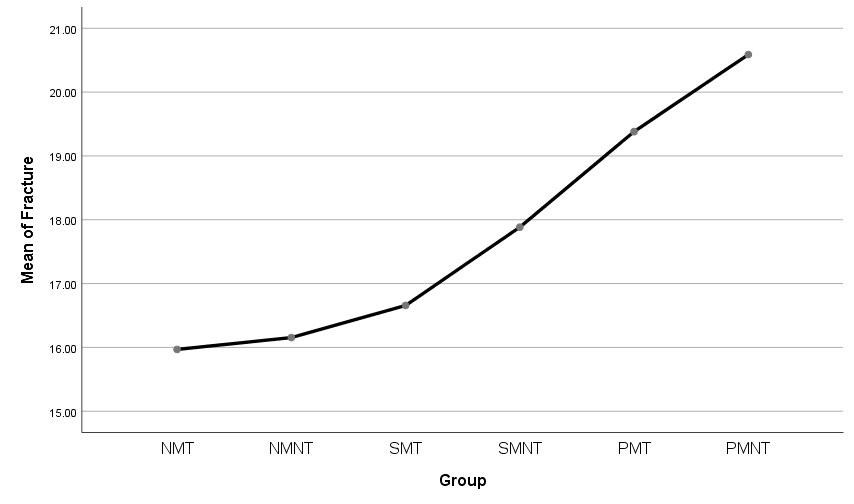
Shear bond strengths in the studied groups.

Figure 3 presents the comparison of the six study groups in terms of the shear bond strength. As shown, the highest shear bond strength was observed in the samples treated with aluminum oxide and primer without thermocycling and masticatory cycle, and the lowest shear bond strength was related to the polished samples with thermocycling and masticatory cycles (P=0.001). Furthermore, almost half of the samples fractured, exhibiting cohesive failure while the other half fractured, exhibiting adhesive failure.

## Discussion

In the present study, the research hypothesis was rejected. Comparison of the groups indicated that the highest shear bond strength was related to the samples treated with aluminum oxide and primer, and the lowest shear bond strength was related to the polished samples (P=0.001).

Surface treatment aims to clean the surface of contamination and debris and increase the shear bond strength.^16^

Air-particle abrasion is a prerequisite for achieving sufficient shear bond strength between the porcelain and high-strength ceramics, reinforced with aluminum or zirconia.^17,25^

The mechanism of bonding the veneering porcelain to zirconia is unknown, and it is claimed that this bonding is merely micromechanical, so that the wettability and superficial roughness factors are reported in some studies.^26^

Lack of porcelain supported with zirconia core and poor bonding between the porcelain and zirconia are considered as the main reasons for this bonding fracture. Aluminum surface treatment increases the bonding surface and surface roughness, leading to bond strength.^27^

According to Aboushelib et al^28^ and Musharraf et al^29^ surface treatment, depending on the type of zirconia, has a different effect on the bond strength. Thus, treatment with a primer is more consistent with the zirconia type used in this study, leading to increased bond strength compared to the other groups. Consistent with a study by Aboushelib et al,^30^ the compatibility of veneering porcelain with surface treatment and zirconia type is vital in the bond strength.

Differences between the results of the present study and some other studies are related to the relative incompatibility of zirconia and veneering porcelain, the different conditions of studies and inherent weakness of some experiments, such as the shear bond strength test. In this regard, there are no standard methods for the bond strength between zirconia and porcelain. Some companies only produce zirconium; therefore, other brands should be used inevitably for the veneering porcelain, which can be regarded as the source of differences.

Kasraee et al^31^ compared the microtensile bond strength of three types of conventional all-ceramic systems and applied two types of surface treatment, with one based on the manufacturer's instructions and another based on using the polished surface. Based on the results, the bond strength of the core‒veneer in Cercon was significantly lower than that of Empre SS2, although the difference with Vita Mark II was not significant. In addition, the surface treatment failed to play a significant role in the strength of the bond since polishing of the core failed to reduce and improve the bond strength. Concerning the liner, they suggested that it should be used in the Cercon system since it increases the bond strength almost two times. The fracture type in Cercon and Vita was mainly at the core‒veneer interface (adhesive). However, half of the fractures were adhesive, and the rest were cohesive in the present study. The results indicated the importance of following the manufacturer's instructions and using its proposed surface treatment, which resulted in doubling the bond strength. However, the same surface treatment even reduced the bond strength in the other type of zirconia. Therefore, the positive results of the interrelation between surface roughness and bond strength in the present study might be due to better compatibility of zirconia with the air abrasion method, and crystallographic and microscopic changes occurring at the surface, not necessarily the direct effect of increased surface roughness of zirconia. Hence, following the manufacturer's instructions is an important factor in increasing the bond strength.

In the present study, the shear bond strength test was used to measure bond strength. This test is the most common test used in different studies, but one of the problems in this test is related to its large dispersion, leading to a large standard deviation of data.^32^ In the study of Aboushelib et al,^33^ the microtensile bond strength and shear bond strength tests were evaluated by which the microtensile bond strength test indicated even more consistent results.

In the study of Vasques et al,^34^ which used a protocol very similar to the present study concerning the application of thermocycling and masticatory cycle, the results were consistent with those of the present study, in which thermocycling did not affect the shear bond strength of zirconia core to porcelain veneer.

The limitations of this study included the lack of investigation and comparison of other surface preparations — no use of the veneer from the same zirconia core manufacture

## Conclusion

Based on the results, treatment with aluminum oxide and primer increased the shear bond strength of zirconia core to porcelain veneer. Thermocycling and masticatory cycle did not significantly reduce the shear bond strength in the three study groups.

## Competing Interests

The authors declare no competing interests with regards to the authorship and/or publication of this article.

## Authors’ Contributions

TG and EM designed the study. MM performed the experiments and drafted the manuscript. All the authors critically revised the manuscript for the intellectual content. All the authors have read and approved the final manuscript.

## Acknowledgments

This study was a part of a PhD thesis and supported by a grant from Tabriz University of Medical Sciences. The authors would like to thank the Dental and Periodontal Research Center of Tabriz University of Medical Sciences.

## Funding

This research was carried out by financial support from the Dental and Periodontal Research Center, Tabriz University of Medical Sciences.

## Ethics Approval

This research was approved by the Research Ethics Committee of Tabriz University of Medical Sciences in 2019.
